# Cardiac and hepatic hydatid cyst in a child with chest pain

**DOI:** 10.1590/0037-8682-0131-2021

**Published:** 2021-04-28

**Authors:** Ismet Mirac Cakir, Serdar Aslan, Tumay Bekci

**Affiliations:** 1 Giresun University, Faculty of Medicine, Department of Radiology, Giresun, Turkey.

**FIGURE 1:**
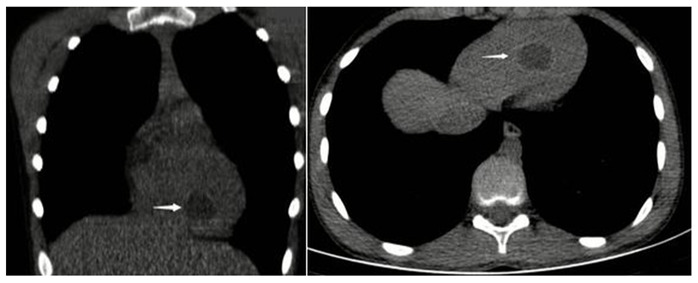
Coronal and axial computed tomography. The cystic lesion is adjacent to the interventricular septum in the heart’s left ventricle (arrow).

**FIGURE 2:**
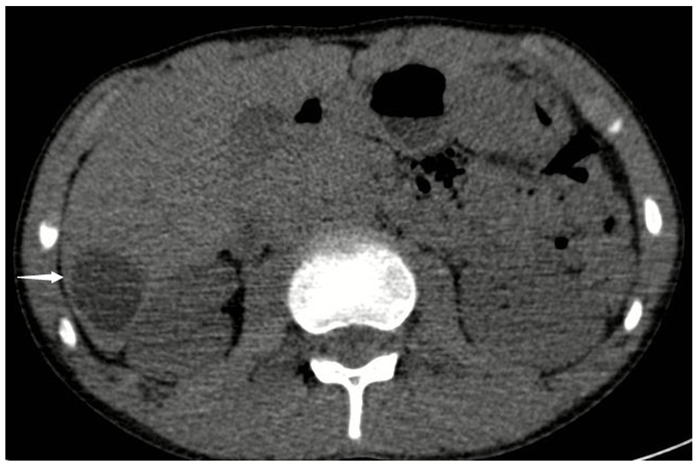
Axial computed tomography. Cystic lesion in liver segment 6 (arrow).

A 12-year-old man with a history of progressive dyspnea and chest pain was admitted to our emergency department. On chest computed tomography (CT), a cyst was observed in the left ventricle of the heart, adjacent to the interventricular septum (Figure 1, arrow). Further, on abdominal CT, a cystic lesion was observed in the liver segment 6 (Figure 2, arrow). Eosinophilia was detected in the laboratory findings. The indirect hemagglutination test (IHA) for the determination of antihydatide cyst antibody was positive (1/3200 IU). CT findings of the patient were consistent with cardiac and hepatic hydatid cyst. The diagnosis of a hydatid cyst was thus surgically confirmed.

Hydatid cyst mostly occurs in the liver and lung. Cardiac involvements are rare. CT is benefical in localizing and diagnosing hydatid cysts. Symptoms of the cardiac hydatid disease range from asymptomatic to life-threatening course. As with our patient, it can occur with chest pain and shortness of breath. Moreover, recurrent syncope attacks associated with palpitations and underlying heart arrhythmias may occur as accompanying symptoms. Cardiac hydatid cyst rupture can cause pulmonary embolism or stroke^1^. Early and correct diagnosis is important, and hydatid cyst should be considered in the differential diagnosis of cystic masses, especially in regions where the disease is endemic.
